# Changes in TCA cycle and TCA cycle-related metabolites in plasma upon citric acid administration in rats

**DOI:** 10.1016/j.heliyon.2021.e08501

**Published:** 2021-12-04

**Authors:** Yurie Hara, Satoshi Kume, Yosky Kataoka, Nakamichi Watanabe

**Affiliations:** a Department of Advanced Food Sciences, College of Agriculture, Tamagawa University, Tokyo, Japan; b Laboratory for Pathophysiological and Health Science, RIKEN Center for Biosystems Dynamics Research, Kobe, Japan; c Center for Health Science Innovation, Osaka City University, Osaka, Japan; d Laboratory for Cellular Function Imaging, RIKEN Center for Biosystems Dynamics Research, Hyogo, Japan; e Multi-modal Microstructure Analysis Unit, RIKEN-JEOL Collaboration Center, RIKEN Baton Zone Program, Hyogo, Japan; f Department of Health Science, Faculty of Food and Health Sciences, Showa Women's University, Tokyo, Japan

**Keywords:** Citric acid, Fatigue, Tricarboxylic acid cycle, Amino acid, Anaplerotic substrates, Metabolome analysis

## Abstract

Recent studies have reported that plasma levels of tricarboxylic acid (TCA) cycle metabolites and TCA cycle-related metabolite change in patients with chronic fatigue syndrome (CFS) and in healthy humans after exercise. Exogenous dietary citric acid has been reported to alleviate fatigue during daily activities and after exercise. However, it is unknown whether dietary citric acid affects the plasma levels of these metabolites. Therefore, the present study aimed to investigate the effects of exogenously administered citric acid on TCA cycle metabolites and TCA cycle-related metabolites in plasma. Sprague-Dawley rats were divided into control and citric acid groups. We evaluated the effect of exogenous dietary citric acid on the plasma TCA cycle and TCA cycle-related metabolites by metabolome analysis using liquid chromatography-tandem mass spectrometry (LC-MS/MS). TCA cycle metabolites, including plasma citrate, cis-aconitate, and isocitrate, were significantly elevated after exogenous administration of citric acid. Anaplerotic amino acids, which are converted to TCA cycle metabolites, such as serine, glycine, tryptophan, lysine, leucine, histidine, glutamine, arginine, isoleucine, methionine, valine, and phenylalanine, also showed significantly elevated levels. Citric acid administration significantly increased the levels of initial TCA cycle metabolites in the plasma. This increase after administration of citric acid was shown to be opposite to the metabolic changes observed in patients with CFS. These results contribute novel insight into the fatigue alleviation mechanism of citric acid.

## 1 Introduction

Dietary citric acid intake alleviates fatigue [1]. Previous randomized, double-blind, placebo-controlled, parallel group studies reported that citric acid alleviates fatigue in daily activities [2, 3]. Furthermore, intake of citric acid before exercise reportedly attenuates physical fatigue and the feeling of fatigue [4, 5, 6, 7].

However, the underlying mechanisms of fatigue alleviation induced by dietary citric acid are currently unknown, although the following hypotheses have been proposed to explain these mechanisms: 1) Sodium citrate reduces the blood pH level, which is believed to reduce muscle contraction during exercise; that is, citric acid serves as a buffering agent. However, there is no evidence supporting the relationship between the reduction in blood pH and alleviation of fatigue [8, 9]. Furthermore, free citric acid has been reported to alleviate fatigue [1, 2, 3, 4, 5]. 2) Citric acid suppresses the accumulation of blood lactate [4, 10], as citric acid produced in the tricarboxylic acid (TCA) cycle inhibits phosphofructokinase [11, 12], which in turn suppresses excess accumulation of blood lactate [10]. However, lactate has recently been reported to be an energy source, not a cause of fatigue [13, 14] 3) Third, because citric acid is an intermediate metabolite in the TCA cycle and it is important for efficient energy production, exogenous dietary citric acid may also activate the TCA cycle. However, the carrier translocating citric acid from the cytoplasm to the mitochondria has not yet been reported. Therefore, dietary citric acid has a markedly low possibility of participating in the TCA cycle [15].

Recent studies have reported that plasma levels of TCA cycle metabolites are reduced in patients with chronic fatigue syndrome (CFS) [16]. In addition, in an animal model of fatigue, the plasma levels of TCA cycle metabolites and TCA cycle-related metabolites have been shown to be altered by fatigue load [17]. TCA cycle-related metabolites are anaplerotic substrates that are converted to TCA cycle metabolites. Consequently, the underlying mechanism of fatigue alleviation induced by dietary citric acid intake may involve alterations in TCA cycle-related metabolites. However, it is unknown whether dietary citric acid influences the plasma levels of TCA cycle and TCA cycle-related metabolites. Therefore, in the present study, we examined the effects of dietary citric acid intake on TCA cycle-related metabolites via metabolome analysis in rat plasma, using liquid chromatography-tandem mass spectrometry (LC-MS/MS).

## 2 Materials and methods

### 2.1 Animals and experimental design

Eight-week-old male Sprague-Dawley rats, a widely used model for fatigue [17], were purchased from Charles River Laboratories (Yokohama, Japan). The rats were housed in individual plastic cages at 22 ± 2 °C, with a 12:12-h light-dark cycle (light from 6 a.m. to 6 p.m.). Rats were maintained for 1 week on a commercial non-purified diet (CRF-1, Charles River Laboratories) and water *ad libitum*. The rats were then divided into two groups with equal mean body weight (n = 8 rats per group; statistical power = 90.8%; significance level = 5%; effect size = 1.35), namely the control and citric acid groups. The mean body weights of the rats in the two groups were equal. The rats were fasted for 6 h, after which distilled water (control) or citric acid (FUJIFILM Wako Pure Chemical Corporation, Osaka, Japan) solution (200 mg/1.5 mL/300 g body weight) was administered intragastrically using a probe. This dose was selected based on a human study related to the performance benefit of citric acid (150–270 mg/300 g body weight). Two and a half hours after administration, the rats were euthanized via blood withdrawal from the abdominal aorta under isoflurane anesthesia. The duration of 2.5 h, from the citric acid administration to euthanasia was set to allow an adequate duration to alter plasma metabolites because plasma citrate levels were high from 30 min to 2.5 h after citric acid administration. Blood samples were collected in vacuum blood collection tubes (7 mL) containing EDTA-2Na (Terumo Corporation, Tokyo, Japan), and plasma samples were obtained via centrifugation at 1,900 ×*g* at 4 °C for 10 min. The samples were immediately frozen in liquid nitrogen and stored at −80 °C for approximately 3 weeks until metabolite extraction. All analyses were performed in an unblinded manner. All animal experiments were performed in accordance with the guidelines approved by the Experimental Animal Ethics Committee of Showa Women's University (approval number: 15-12).

### 2.2 Metabolite extraction

For extraction of plasma metabolites, 50 μL plasma samples were mixed with 450 μL methanol containing internal standards (20 μM each [final concentrations: 18 μM] of _L_-methionine sulfone, _D_-camphor-10-sulfonic acid, and 2-morpholinoethanesulfonic acid). The samples were mixed with 500 μL chloroform and 200 μL ultra-pure water and centrifuged at 14,000 ×*g* at 4 °C for 10 min. Subsequently, the obtained supernatant aqueous layer was filtered via centrifugation through a 3-kDa cutoff filter (Merck Millipore Amicon Ultra, Darmstadt, Germany) at 12,000 ×*g* at 4 °C for 60 min. The filtered samples were concentrated via centrifugation at 35 °C for 1.5 h. The dried samples were dissolved in 25 μL of ultrapure water for LC-MS/MS analysis. By performing the LC-MS/MS method using a quadrupole mass spectrometer, the measurement sensitivity of organic acids and other substances is higher than that by TOF-MS. Furthermore, with the advent of new separation columns, LC measurements of organic acids and amino acids have become possible, and the new separation system was used in this study.

### 2.3 LC-MS/MS conditions for anionic and cationic metabolite analysis

LC/MS-grade solvents, ultra-pure water, and acetonitrile were obtained from FUJIFILM Wako Pure Chemical Corporation, Ltd. (Osaka, Japan); formic acid (99%) was obtained from FUJIFILM Wako Pure Chemical Corporation, Ltd., Osaka, Japan; ammonium formate (99%), Sigma-Aldrich, St. Louis, MO, USA. Research-grade metabolite standards were also used. A mixed solution of the standards was prepared by diluting the stock solutions with ultrapure water immediately before LC/MS analysis.

LC-MS analysis was performed using an LC/MS/MS system, a QTRAP 4500 (AB Sciex, Tokyo, Japan), a Shimadzu Nexera X2 high-performance liquid chromatography (HPLC) pump with an electrospray ionization TurboIonSpray system (AB Sciex, Tokyo, Japan). The electrospray ionization spray voltage was 4500 V in positive mode and -4500 V in negative mode. Nitrogen was used as the curtain gas at 30 psi and as the collision gas at 4, and the nebulizer gas and turbo gas at 50 psi and 80 psi, respectively; capillary temperature, 400 °C.

For anion analysis of organic acids, a Scherzo SM-C18 column (dimensions: 50 mm × 2 mm, Imtakt Corp., Kyoto, Japan) was used. The LC solvents used for anion analysis were solvent A: 100:0.3 = ultra-pure water: formic acid; Solvent B: 100:2 = acetonitrile: formic acid. The linear gradient for LC was as follows: t = 0, 0% B; t = 7 min, 15% B; t = 10 min, 50% B; t = 16.9 min, 50% B; t = 17 min, 0% B; t = 20 min, 0% B. LC conditions included an autosampler temperature of 4 °C, column temperature of 40 °C, injection volume of 0.25% or 5 μL, and solvent flow rate of 250 μL/min. The multiple reaction monitoring (MRM) parameters and retention times of the negative ion mode LC-MS study were listed in Supplementary Table 1 (Table S1). A representative MRM chromatograph for organic acids was shown in Supplementary Figure 1 (Figure S1). In this chromatograph, isomers such as citrate and isocitrate were separated as different peaks (Figure S1). For cation analysis of amino acids, an Intrada Amino Acid column (Dimensions: 100 mm × 2 mm, Imtakt Corp., Kyoto, Japan) was used. The LC solvents used for cation analysis were solvent A: 40:360:100 = 1 M ammonium formate aqueous solution: ultra-pure water: acetonitrile; solvent B: 100:0.3 = acetonitrile: formic acid. The linear gradient for LC was as follows: t = 0, 80% B; t = 3.3 min, 80% B; t = 11.7 min, 0% B; t = 15 min, 0% B; t = 15.1 min, 80% B; t = 17.5 min, 80% B. LC conditions included autosampler temperature 4 °C, column temperature 40 °C, injection volume 0.2 or 5 μL, and solvent flow rate 300 μL/min. The MRM parameters and retention times of the positive ion mode LC-MS study were listed in Supplementary Table 2 (Table S2). A representative MRM chromatograph for amino acids was shown in Supplementary Figure 2 (Figure S2). In this chromatograph, isomers such as leucine and isoleucine were separated as different peaks (Figure S2).

### 2.4 LC/MS data processing, bioinformatics, and statistical analysis

LC/MS/MS peak data were obtained, and peaks were analyzed using MultiQuant™ 2.0 (AB Sciex, Tokyo, Japan). Using the R language platform (R Foundation for Statistical Computing, http://www.r-project.org), each metabolite peak was calculated as the molar concentration from an external calibration curve method of metabolite standards, and data were normalized on the basis of theoretical concentrations of internal standards (methionine sulfone and CSA). Methionine sulfone and CSA are among the most commonly used internal standards, which are relatively stable when stored and show little change in concentrations even after long-term measurements. Statistical analysis of the metabolome data was performed using GraphPad Prism7 (GraphPad Software, CA, USA). After performing the normality test on the data, differences (*P* < 0.05, indicating statistical significance) between rats in the control and citric acid groups were evaluated using the Student's t-test (two-tailed).

### 2.5 Plasma pyruvate assay and statistical analysis

Plasma pyruvate concentration was measured using a pyruvate colorimetric assay kit (BioVision, San Diego, CA, USA) according to the manufacturer's instructions. Data are expressed as the mean ± standard error (SE). After performing the normality test on the data, the Student's t-test was used to examine the differences between rats in the two groups. Results were considered significant at *P* < 0.05.

## 3 Results

Plasma samples of rats in the control and citric acid groups were analyzed using LC-MS/MS, and 10 anionic metabolites and 43 cationic metabolites were identified and quantified (Tables 1 and 2).

**Table 1:** Plasma organic acid concentrations measured by anion analysis in rats.

Metabolites (μM)	Control	Citric acid	P value
Lactate	2129 ± 120	1914 ± 163	0.31
Citrate∗∗∗	61.7 ± 4.1	126 ± 12.7	P < 0.001
cis-Aconitate∗∗∗	2.9 ± 0.2	6.0 ± 0.6	P < 0.001
Isocitrate∗∗∗	5.7 ± 0.3	9.2 ± 0.7	P < 0.001
α-ketoglutarate	23.2 ± 1.2	26.9 ± 1.3	0.054
Succinate	4.9 ± 0.4	4.2 ± 0.3	0.19
Fumarate	1.4 ± 0.1	1.5 ± 0.1	0.36
Malate	31.8 ± 3.1	37.3 ± 2.4	0.18
Pyroglutamate∗∗	26.2 ± 2.8	13.6 ± 2.7	0.006
Pantothenate	0.8 ± 0.1	0.6 ± 0.0	0.19

Data are expressed as mean ± standard error (SE) of eight rats.

Values are significantly different from that of the control group at ∗∗P < 0.01, ∗∗∗P < 0.001.

**Table 2:** Plasma amino acids concentrations measured by cation analysis.

Metabolites (μM)	Control	Citric acid	P value
Betaine	168 ± 10	156 ± 10	0.42
Phenylalanine∗∗	34.0 ± 1.3	39.5 ± 0.7	0.003
Kynurenine∗∗∗	3.0 ± 0.4	6.6 ± 0.7	P < 0.001
Tryptophan∗	42.8 ± 2.1	49.8 ± 1.1	0.012
Leucine∗∗	90.0 ± 4.9	106 ± 3	0.009
Creatinine∗∗	21.3 ± 0.9	17.0 ± 0.5	0.001
Dimethylglycine	6.7 ± 0.5	6.1 ± 0.4	0.35
Isoleucine∗∗∗	44.3 ± 2.7	57.6 ± 1.3	P < 0.001
Methionine∗∗	34.9 ± 1.1	44.2 ± 0.6	P < 0.001
Proline	128 ± 8	137 ± 7	0.44
Tyrosine	42.3 ± 1.9	43.6 ± 2.1	0.65
Valine∗∗	125 ± 7	149 ± 3	0.004
Carnitine	64.1 ± 7.2	54.4 ± 4.6	0.27
Hydroxyproline	67.9 ± 6.8	63.1 ± 8.2	0.66
Choline	7.9 ± 0.2	8.0 ± 0.2	0.92
Creatine∗∗∗	131 ± 17	307 ± 34	P < 0.001
Sarcosine	499 ± 23	497 ± 17	0.95
Threonine	258 ± 23	333 ± 30	0.066
Glutamate∗	93.1 ± 4.1	80.4 ± 3.5	0.032
Guanidinoacetate	4.6 ± 0.4	4.5 ± 0.4	0.96
Aspartate	7.0 ± 0.5	7.0 ± 0.5	0.96
Glycine∗∗	278 ± 13	357 ± 18	0.003
Glutamine∗	720 ± 28	820 ± 26	0.021
Serine∗∗	188 ± 11	238 ± 9	0.003
β-alanine	3.4 ± 0.2	3.8 ± 0.2	0.21
Citrulline	141 ± 9	121 ± 5	0.074
Acetyllysine	0.16 ± 0.01	0.17 ± 0.02	0.68
γ-aminobutyric acid	0.21 ± 0.01	0.23 ± 0.01	0.54
Glycylglycine	0.08 ± 0.01	0.09 ± 0.01	0.69
Cystine∗	74.1 ± 6.7	112.6 ± 11.1	0.010
Histidine∗∗∗	38.1 ± 4.2	67.0 ± 1.2	P < 0.001
Hydroxylysine∗	1.0 ± 0.1	1.2 ± 0.1	0.016
Lysine∗∗∗	260 ± 11	380 ± 11	P < 0.001
Ornithine∗∗∗	23.4 ± 1.1	32.1 ± 1.5	P < 0.001
Carnosine	1.2 ± 0.1	1.4 ± 0.1	0.16
Arginine∗	100 ± 9	124 ± 4	0.029
Acetylglycine	152 ± 26	148 ± 17	0.90
Allantoin	153 ± 11	169 ± 10	0.30
Taurine	177 ± 14	160 ± 13	0.38
Glutathione	133 ± 11	140 ± 15	0.70
Hypotaurine	162 ± 19	147 ± 5	0.46
3-aminobutyric acid	155 ± 27	159 ± 12	0.89
Asparagine∗∗∗	126 ± 5	197 ± 10	P < 0.001

Data are expressed as mean ± standard error (SE) of eight rats.

Values are significantly different from that of the control group at ∗P < 0.05, ∗∗P < 0.01, ∗∗∗P < 0.001.

**Figure 1:**
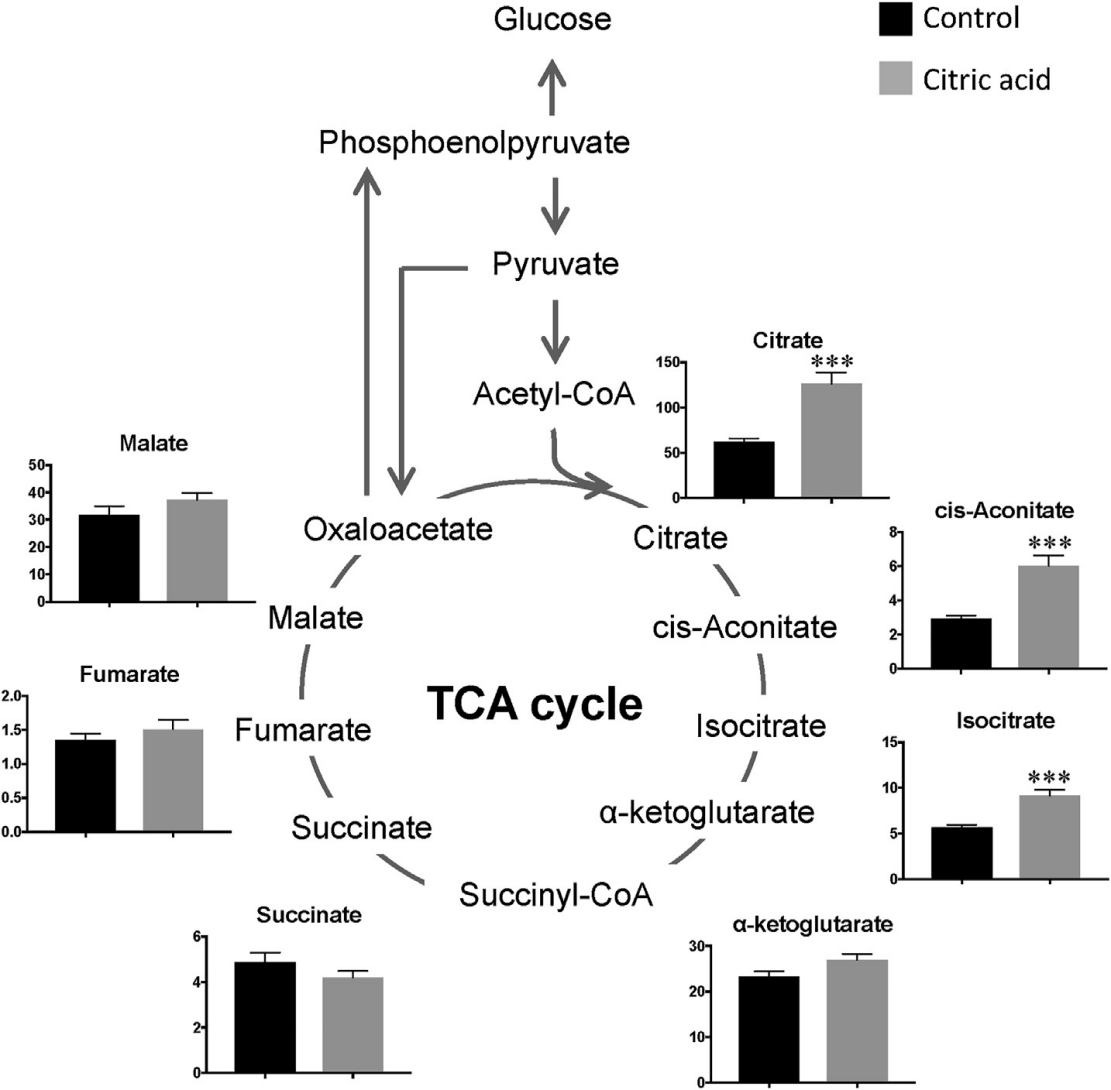
Plasma levels of TCA cycle metabolites in rats measured by LC-MS/MS analysis. Black and gray bars indicate the control and citric acid groups, respectively. Data are expressed as the mean ± standard error (SE) of the eight rats. Values are significantly different from those of the control group at ∗∗∗P < 0.001.

Of the anionic metabolites, 10 metabolites were quantified, of which seven were TCA cycle metabolites (Figure 1). Plasma citrate, cis-aconitate, and isocitrate levels were significantly higher in rats in the citric acid group than in control rats (*P* < 0.001), whereas plasma α-ketoglutarate levels tended to be higher in rats in the citric acid group than in control rats (*P* = 0.054). Plasma succinate, fumarate, and malate levels did not differ significantly between rats in the control and citric acid groups.

**Figure 2:**
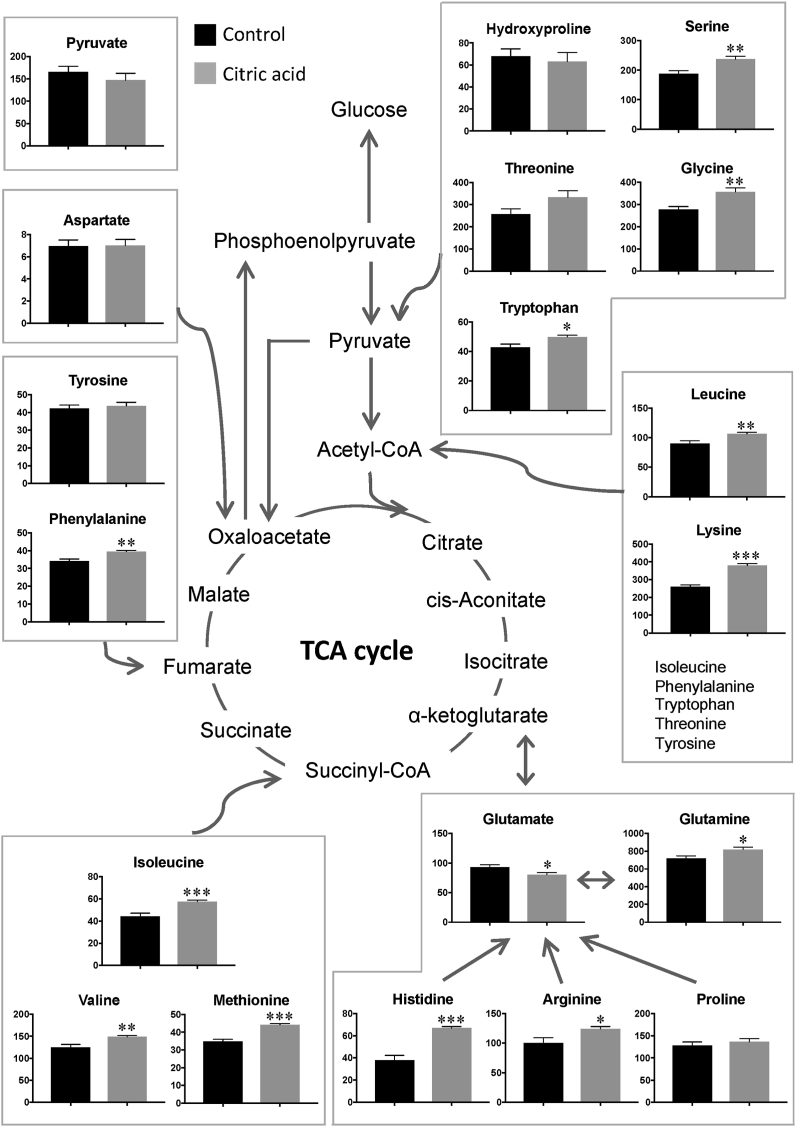
Plasma levels of anaplerotic amino acids of TCA cycle metabolites in rats measured by LC-MS/MS analysis. Black and gray bars indicate the control and citric acid groups, respectively. Plasma pyruvate levels (upper left corner in this figure) were measured using a pyruvate colorimetric assay kit. Data are expressed as the mean ± standard error (SE) of the eight rats. Values are significantly different from those of the control group at ∗P < 0.05, ∗∗P < 0.01, and ∗∗∗P < 0.001.

Of the cationic metabolites, 43 metabolites were quantified, of which 18 were anaplerotic substrates of TCA cycle metabolites (Figure 2). Anaplerotic substrates complement TCA cycle metabolites to maintain TCA cycle homeostasis. Hydroxyproline, serine, threonine, glycine, and tryptophan are anaplerotic substrates that are converted to pyruvate. In this study, plasma serine, glycine, and tryptophan levels were significantly higher in rats in the citric acid group than in rats in the control group. Plasma hydroxyproline and threonine levels did not differ significantly between rats in the two groups. Glutamate, glutamine, histidine, arginine, and proline are anaplerotic substrates that are converted to α-ketoglutarate. Plasma glutamine, histidine, and arginine levels were significantly higher in rats in the citric acid group than in control rats, while glutamate levels were significantly lower in rats in the citric acid group than in control rats, and proline levels remained unchanged. Isoleucine, valine, and methionine are anaplerotic substrates that are converted to succinyl-CoA, and their plasma levels were significantly higher in rats in the citric acid group than in control rats. Phenylalanine and tyrosine are anaplerotic substrates that are converted to fumarate. Plasma phenylalanine levels were significantly higher in rats in the citric acid group than in control rats, while tyrosine levels remained unchanged. Aspartate is an anaplerotic substrate that is converted to oxaloacetate, and its plasma levels remained unchanged between rats in the two groups. Leucine, isoleucine, lysine, phenylalanine, tryptophan, threonine, and tyrosine are anaplerotic substrates and are converted to acetyl-CoA, and their plasma levels (except for threonine and tyrosine) were significantly higher in rats in the citric acid group than in control rats, whereas plasma threonine and tyrosine levels did not differ significantly.

Because plasma pyruvate levels were not quantifiable by LC-MS/MS, they were determined using a pyruvate colorimetric assay kit (Bio Vision, CA, USA), which showed no significant difference between rats in the two groups (upper left corner in Figure 2).

## 4 Discussion

Dietary citric acid intake results in fatigue alleviation during daily activities and after exercise [2, 3, 4, 5, 6, 7]; nevertheless, the underlying mechanism is currently unknown. A recent study reported that levels of initial TCA cycle metabolites in plasma, namely, citrate, cis-aconitate, and isocitrate decrease in CFS patients [16]. In addition, TCA cycle-related metabolites were altered in a rat model of fatigue [17]. However, it is unclear whether dietary citric acid intake affects the TCA cycle and TCA cycle-related metabolites. In the present study, we determined the effects of exogenous dietary citric acid intake on the TCA cycle and TCA cycle-related metabolites in rat plasma, using LC-MS/MS analysis, thereby revealing the following effects of citric acid administration: 1) initial TCA cycle metabolites, namely citrate, cis-aconitate, and isocitrate, were significantly higher, whereas α-ketoglutarate tended to be high; 2) levels of anaplerotic amino acids, which are converted to TCA cycle metabolites, namely serine, glycine, tryptophan, lysine, leucine, histidine, glutamine, arginine, isoleucine, methionine, valine, and phenylalanine, increased significantly. The increase in plasma citrate, cis-aconitate, and isocitrate after the administration of citric acid was shown to be opposite to the metabolic changes in CFS. Therefore, these results suggest that intake of citric acid has the potential to alleviate fatigue.

The increase in initial TCA cycle metabolites, that is citrate, cis-aconitate, isocitrate, and α-ketoglutarate, can be explained by three potential hypotheses. The first hypothesis is that dietary citric acid enters the cytoplasm and subsequently translocates to the mitochondria. However, assuming this intracellular translocation, plasma levels of not only citrate, cis-aconitate, isocitrate, and α-ketoglutarate, but also succinate, fumarate, and malate are expected to increase after citric acid administration. Furthermore, although the citric acid in the mitochondria can be translocated to the cytoplasm through the mitochondrial citrate carrier SLC25A1, which promotes the translocation of citrate/isocitrate across the mitochondria, in exchange for the translocation of cytoplasmic malate [18, 19, 20, 21], the transport of citric acid from the cytoplasm to the mitochondria has not yet been reported. In addition, we have revealed that dietary citric acid did not upregulate the expression of genes related to the TCA cycle in the liver and skeletal muscle of mice [22, 23]. Accordingly, the possibility that dietary citric acid can translocate from the cytoplasm to the mitochondria is considerably low.

The second hypothesis is that the increase in initial TCA cycle metabolites is due to an increase in anaplerotic substrates such as amino acids, which can be converted to pyruvate. Because the TCA cycle is central to energy metabolism, adequate maintenance of TCA cycle intermediates is critical [24]. Therefore, there is a system to replenish these metabolites (*anaplerosis*) from glucose, fatty acids, and amino acids [24]. For instance, amino acids including hydroxyproline, serine, threonine, glycine, and tryptophan can be converted to pyruvate; thereafter, pyruvate is used to supply TCA cycle metabolites. That is, an increase in pyruvate levels presumably leads to an increase in TCA cycle metabolites. In the present study, citric acid administration led to an increase in plasma serine, glycine, and tryptophan levels, which can be converted to pyruvate. However, the plasma pyruvate level remained unchanged after citric acid administration. Therefore, it was suggested that an increase in initial TCA cycle metabolites in rats in the citric acid group was not due to an increase in amino acids that can be converted to pyruvate. In addition, the plasma levels of succinate and fumarate did not increase with citric acid administration, although isoleucine, methionine, valine, and phenylalanine levels increased significantly. Isoleucine, methionine, and valine are anaplerotic substrates that can be converted to succinyl-CoA, while phenylalanine is converted to fumarate. Therefore, these amino acids, namely serine, glycine, tryptophan, isoleucine, methionine, valine, and phenylalanine, have a lower possibility of serving as anaplerotic substrates to replenish TCA cycle metabolites. Citric acid administration led to an increase in plasma levels of 12 amino acids; however, the underlying mechanism is unknown, although plasma amino acids are thought to be supplied from skeletal muscle and other organs. These amino acids are possible substrates of gluconeogenesis, as citric acid administration has been reported to increase plasma glucose levels in mice [22, 23]. Further studies are needed to clarify the mechanism by which citric acid administration increases plasma amino acid levels. As described earlier, the increase in initial TCA cycle metabolites induced by dietary citric acid has a low possibility of being induced by the replenishment of amino acids.

The third hypothesis, which, we believe, is the most convincing hypothesis, indicates that an increase in initial TCA cycle metabolites, citrate, cis-aconitate, isocitrate, and α-ketoglutarate, on citric acid administration occurred in the cytoplasm. Aconitase converts citrate into cis-aconitate and isocitrate [25]. Isocitrate dehydrogenase converts isocitrate to α-ketoglutarate. Both enzymes are present not only in the mitochondria but also in the cytoplasm [25, 26], although these enzymes have been known as the TCA cycle-related enzymes. In addition, as previously mentioned in the first hypothesis of the "Discussion" section, it is unlikely that dietary citric acid enters the mitochondria from the cytoplasm as the synthesis of glycogen, which occurs in the cytoplasm, is promoted in the liver and skeletal muscle following citric acid administration [27]. Therefore, an increase in the levels of initial TCA cycle metabolites after citric acid administration may have occurred in the cytoplasm but not in the mitochondria.

This study has three limitations. First, the present study could not directly prove the phenomenon of the increase in initial TCA cycle metabolites occurring in the cytoplasm and/or mitochondria. Second, the phenomenon was confirmed at only one time point, that is, 2.5 h after citric acid administration. Third, this study was performed using rats without fatigue. Therefore, future studies using fatigue-loaded rats, such as rats after exercise, and at different time points, are needed to clarify the association between citric acid administration and fatigue alleviation and the underlying mechanisms.

## 5 Conclusion

We examined the effects of dietary citric acid intake on TCA cycle-related metabolites via metabolome analysis in rat plasma. Citric acid administration significantly increased the levels of initial TCA cycle metabolites such as citrate, cis-aconitate, and isocitrate, in the cytoplasm but not in the mitochondria. If dietary citric acid alleviates fatigue, as reported previously, it could serve as a key cytoplasmic factor. In addition, citric acid administration has the potential to alleviate the symptoms in CFS patients because the plasma levels of initial TCA cycle metabolites have been reported to be low in CFS patients.

## Declarations

### Author contribution statement

Yurie Hara: Conceived and designed the experiments; Performed the experiments; Analyzed and interpreted the data; Contributed reagents, materials, analysis tools or data; Wrote the paper.

Satoshi Kume: Performed the experiments; Analyzed and interpreted the data; Contributed reagents, materials, analysis tools or data; Wrote the paper.

Yosky Kataokad: Conceived and designed the experiments; Analyzed and interpreted the data; Contributed reagents, materials, analysis tools or data.

Nakamichi Watanabe: Conceived and designed the experiments; Analyzed and interpreted the data; Contributed reagents, materials, analysis tools or data; Wrote the paper.

### Funding statement

This work was supported by a JSPS Grant-in-Aid for JSPS Fellows [grant number 14J07401].

### Data availability statement

Data included in article/supplementary material/referenced in article.

### Declaration of interests statement

The authors declare no conflict of interest.

### Additional information

No additional information is available for this paper.

## Appendix A Supplementary data

Fig_S1.&S2.docx Fig_S1.&S2.docx

Table_S1&S2.docx Table_S1&S2.docx

Table_S3.xlsx Table_S3.xlsx

## References

[1] de Salles Painelli V., Lancha Junior A.H. (2018). Thirty years of investigation on the ergogenic effects of sodium citrate: is it time for a fresh start?. Br. J. Sports Med..

[2] Kajimoto O., Mieda H., Hiramitsu M., Sakaida K., Yasuda T., Sugino T., Kajimoto Y. (2007). The internet investigation about the attenuation of fatigue feeling by taking a drink containing lemon citric acid. Jpn. Pharmacol. Ther..

[3] Kajimoto O., Mieda H., Hiramitsu M., Sakaida K., Sugino T., Kajimoto Y. (2007). Effect of a drink containing lemon citric acid on people frequently feeling fatigue. Jpn. Pharmacol. Ther..

[4] Kono R., Nomura S., Tokuda A., Okuno Y., Fujihira Y., Kamei I., Nakamura M., Utsunomiya H. (2017). Effects of citric acid oral intake before low intensity exercise on blood lactic acid and feeling of fatigue—a randomized, double-blind, placebo-controlled, cross-over study. Jpn. Pharmacol. Ther..

[5] Sugino T., Aoyagi S., Shirai T., Kajimoto Y., Kajimoto O. (2007). Effects of citric acid and _L_-carnitine on physical fatigue. J. Clin. Biochem. Nutr..

[6] Oöpik V., Saaremets I., Medijainen L., Karelson K., Janson T., Timpmann S. (2003). Effects of sodium citrate ingestion before exercise on endurance performance in well trained college runners. Br. J. Sports Med..

[7] McNaughton L., Cedaro R. (1992). Sodium citrate ingestion and its effects on maximal anaerobic exercise of different durations. Eur. J. Appl. Physiol. Occup. Physiol..

[8] Carr A.J., Hopkins W.G., Gore C.J. (2011). Effects of acute alkalosis and acidosis on performance: a meta-analysis. Sports Med..

[9] Lancha Junior A.H., Painelli Vde S., Saunders B., Artioli G.G. (2015). Nutritional strategies to modulate intracellular and extracellular buffering capacity during high-intensity exercise. Sports Med..

[10] Miyake Y., Yamamoto K., Nagasaki M., Nakai N., Murakami T., Shimomura Y. (2001). Influence of lemon juice and citrate on blood lactate concentration after exercise in humans. JJSNFS.

[11] Tornheim K., Lowenstein J.M. (1976). Control of phosphofructokinase from rat skeletal muscle. Effects of fructose diphosphate, AMP, ATP, and citrate. J. Biol. Chem..

[12] Underwood A.H., Newsholme E.A. (1965). Properties of phosphofructokinase from rat liver and their relation to the control of glycolysis and gluconeogenesis. Biochem. J..

[13] Robergs R.A., Ghiasvand F., Parker D. (2004). Biochemistry of exercise-induced metabolic acidosis. Am. J. Physiol. Regul. Integr. Comp. Physiol..

[14] Kitaoka Y., Hoshino D., Hatta H. (2012). Monocarboxylate transporter and lactate metabolism. J. Phys. Fit. Sports Med..

[15] Baldwin J.E., Krebs H. (1981). The evolution of metabolic cycles. Nature.

[16] E. Yamano, M. Sugimoto, A. Hirayama, S. Kume, M. Yamato, G. Jin, et al, Index markers of chronic fatigue syndrome with dysfunction of TCA and urea cycles, Sci. Rep., 6 34990. 10.1038/srep34990 PMC5057083 27725700

[17] Kume S., Yamato M., Tamura Y., Jin G., Nakano M., Miyashige Y., Eguchi A., Ogata Y., Goda N., Iwai K., Yamano E., Watanabe Y., Soga T., Kataoka Y. (2015). Potential biomarkers of fatigue identified by plasma metabolome analysis in rats. PLoS One.

[18] Bisaccia F., De Palma A., Prezioso G., Palmieri F. (1990). Kinetic characterization of the reconstituted tricarboxylate carrier from rat liver mitochondria. Biochim. Biophys. Acta.

[19] Krämer R., Palmieri F. (1989). Molecular aspects of isolated and reconstituted carrier proteins from animal mitochondria. Biochim. Biophys. Acta.

[20] Palmieri F., Monné M. (2016). Discoveries, metabolic roles and diseases of mitochondrial carriers: a review. Biochim. Biophys. Acta.

[21] Palmieri F. (2014). Mitochondrial transporters of the SLC25 family and associated diseases: a review. J. Inherit. Metab. Dis..

[22] Hara Y., Watanabe N. (2013). Effects of dietary citric acid on metabolic indicators and gene expression in the skeletal muscles of fasted mice. Food Nutr. Sci..

[23] Hara Y., Watanabe N. (2015). Fatigue alleviation mechanism of citric acid determined by gene expression analysis in the mouse liver. Food Nutr. Sci..

[24] Owen O.E., Kalhan S.C., Hanson R.W. (2002). The key role of anaplerosis and cataplerosis for citric acid cycle function. J. Biol. Chem..

[25] Krebs H.A., Holzach O. (1952). The conversion of citrate into cis-aconitate and isocitrate in the presence of aconitase. Biochem. J..

[26] Jo S.H., Son M.K., Koh H.J., Lee S.M., Song I.H., Kim Y.O. (2001). Control of mitochondrial redox balance and cellular defense against oxidative damage by mitochondrial NADP^+^-dependent isocitrate dehydrogenase. J. Biol. Chem..

[27] Saitoh S.I., Yoshitake Y., Suzuki M. (1983). Enhanced glycogen repletion in liver and skeletal muscle with citrate orally fed after exhaustive treadmill running and swimming. J. Nutr. Sci. Vitaminol..

