# Supplementary material for: Changes in TCA cycle and TCA cycle-related metabolites in plasma upon citric acid administration in rats
Source: Heliyon. 2021 Dec 4;7(12):e08501. doi: 10.1016/j.heliyon.2021.e08501 (PMC8654791; doi:10.1016/j.heliyon.2021.e08501)
Supplement: Fig_S1.&S2.docx [file mmc1.docx]

Fig. S1. Representative MRM chromatograph of the negative ion mode LC-MS measurement. For anion analysis of organic acids, a Scherzo SM-C18 column (dimensions: 50 mm × 2 mm, Imtakt Corp., Kyoto, Japan) and standard chemicals were used. The MRM parameters were listed in the Table S1.

Fig. S2. Representative MRM chromatograph of the positive ion mode LC-MS measurement. For cation analysis of amino acids, an Intrada Amino Acid column (Dimensions: 100 mm × 2 mm, Imtakt Corp., Kyoto, Japan) and standard chemicals were used. The MRM parameters were listed in the Table S2.
