# Supplementary material for: Changes in TCA cycle and TCA cycle-related metabolites in plasma upon citric acid administration in rats
Source: Heliyon. 2021 Dec 4;7(12):e08501. doi: 10.1016/j.heliyon.2021.e08501 (PMC8654791; doi:10.1016/j.heliyon.2021.e08501)
Supplement: Table_S1&S2.docx [file mmc2.docx]

| Table S1. MRM parameters for negative ion mode LC-MS measurement. | | | | | | | |
| --- | --- | --- | --- | --- | --- | --- | --- |
| Q1  (m/z) | Q3  (m/z) | Retention time  (min) | Metabolites | DP  (V) | EP  (V) | CE  (V) | CXP  (V) |
| 88.93 | 43.10 | 1 | Lactate | -45 | -10 | -16 | -7 |
| 132.87 | 115.00 | 1 | Malate | -45 | -10 | -14 | -5 |
| 190.87 | 72.90 | 1.3 | Isocitrate | -55 | -10 | -28 | -7 |
| 116.87 | 98.90 | 1.3 | Succinate | -45 | -10 | -14 | -5 |
| 127.69 | 84.00 | 1.4 | Pyroglutamate | -60 | -10 | -14 | -9 |
| 190.80 | 110.90 | 1.9 | Citrate | -5 | -10 | -18 | -9 |
| 114.88 | 70.90 | 2.2 | Fumarate | -40 | -10 | -10 | -7 |
| 144.86 | 57.00 | 3.2 | α-ketoglutarate | -5 | -10 | -14 | -5 |
| 217.93 | 88.00 | 3.8 | Pantothenate | -80 | -10 | -18 | -7 |
| 172.81 | 84.90 | 6.7 | *cis*-Aconitate | -5 | -10 | -16 | -7 |
| 230.89 | 79.80 | 14 | CSA (IS) | -125 | -10 | -54 | -7 |
| IS: Internal standard, DP: Declustering potential, EP: Entrance potential, CE: Collision energy, and CXP: collision cell exit potential | | | | | | | |

| Table S2. MRM parameters for positive ion mode LC-MS measurement. | | | | | | | |
| --- | --- | --- | --- | --- | --- | --- | --- |
| Q1  (m/z) | Q3  (m/z) | Retention time  (min) | Metabolites | DP  (V) | EP  (V) | CE  (V) | CXP  (V) |
| 117.882 | 76.1 | 1.4 | Acetylglycine | 36 | 10 | 11 | 12 |
| 158.86 | 61.00 | 1.8 | Allantoin | 51 | 10 | 11 | 6 |
| 195.91 | 100.00 | 2.6 | MES (IS) | 91 | 10 | 29 | 8 |
| 118.01 | 58.00 | 3.3 | Betaine | 46 | 10 | 39 | 16 |
| 165.98 | 120.10 | 3.5 | Phenylalanine | 51 | 10 | 19 | 6 |
| 208.92 | 192.00 | 3.6 | Kynurenine | 1 | 10 | 11 | 10 |
| 204.91 | 146.10 | 3.7 | Trptophan | 46 | 10 | 23 | 8 |
| 132.07 | 86.20 | 3.8 | Leucine | 46 | 10 | 13 | 14 |
| 113.98 | 44.00 | 4 | Creatinine | 16 | 10 | 29 | 12 |
| 103.94 | 44.00 | 4.1 | Dimethylglycine | 41 | 10 | 49 | 12 |
| 132.07 | 69.10 | 4.1 | Isoleucine | 46 | 10 | 23 | 18 |
| 150.00 | 104.00 | 4.3 | Metionine | 41 | 10 | 13 | 28 |
| 116.01 | 70.00 | 4.6 | Proline | 11 | 10 | 19 | 20 |
| 181.93 | 136.20 | 4.6 | Tyrosine | 46 | 10 | 17 | 8 |
| 118.02 | 72.00 | 4.8 | Valine | 46 | 10 | 15 | 20 |
| 125.90 | 107.90 | 5.4 | Taurine | 61 | 10 | 15 | 8 |
| 181.91 | 56.00 | 5.7 | Methionine sulfone (IS) | 11 | 10 | 33 | 8 |
| 162.01 | 103.00 | 5.9 | Carnitine | 61 | 10 | 23 | 16 |
| 132.01 | 86.10 | 5.9 | Hydoxyproline | 56 | 10 | 19 | 6 |
| 104.01 | 60.00 | 6.1 | Choline | 46 | 10 | 23 | 16 |
| 132.02 | 90.00 | 6.1 | Creatine | 36 | 10 | 17 | 6 |
| 89.95 | 44.00 | 6.2 | Sarcosine | 36 | 10 | 15 | 8 |
| 120.01 | 74.00 | 6.2 | Threonine | 6 | 10 | 15 | 20 |
| 307.84 | 178.90 | 6.2 | Glutathione reduced | 56 | 10 | 17 | 12 |
| 109.90 | 91.90 | 6.2 | Hypotaurine | 36 | 10 | 13 | 6 |
| 148.00 | 84.10 | 6.3 | Glutamate | 41 | 10 | 21 | 4 |
| 117.95 | 76.00 | 6.3 | Guanidinoacetate | 41 | 10 | 15 | 4 |
| 103.94 | 44.00 | 6.3 | 3-aminobutyric acid (3AB) | 41 | 10 | 17 | 12 |
| 134.00 | 74.00 | 6.6 | Aspartate | 36 | 10 | 17 | 20 |
| 75.988 | 30.1 | 6.6 | Glycine | 36 | 10 | 17 | 8 |
| 147.01 | 84.00 | 6.7 | Glutamine | 31 | 10 | 21 | 14 |
| 105.98 | 60.10 | 6.8 | Serine | 41 | 10 | 15 | 16 |
| 132.96 | 73.90 | 6.8 | Asparagine | 16 | 10 | 19 | 4 |
| 89.97 | 72.00 | 7.1 | β-Alanine (bAla) | 36 | 10 | 11 | 20 |
| 176.00 | 113.00 | 7.1 | Citrulline | 16 | 10 | 21 | 18 |
| 188.92 | 84.00 | 7.3 | Acetyllysine | 51 | 10 | 29 | 6 |
| 103.947 | 87 | 7.3 | γ-aminobutyric acid (GABA) | 31 | 10 | 13 | 6 |
| 133.02 | 76.00 | 7.9 | Glycylglycine | 1 | 10 | 13 | 12 |
| 240.78 | 151.80 | 8.2 | Cystine | 11 | 10 | 19 | 10 |
| 155.99 | 110.10 | 11.8 | Histidine | 46 | 10 | 19 | 6 |
| 162.92 | 128.00 | 12.5 | Hydroxylysine | 31 | 10 | 15 | 8 |
| 147.02 | 84.20 | 12.6 | Lysine | 16 | 10 | 21 | 6 |
| 133.05 | 70.10 | 12.7 | Ornithine | 36 | 10 | 21 | 20 |
| 226.94 | 110.10 | 12.8 | Carnosine | 66 | 10 | 29 | 6 |
| 175.00 | 70.00 | 13.5 | Arginine | 31 | 10 | 27 | 12 |
| IS: Internal standard, DP: Declustering potential, EP: Entrance potential, CE: Collision energy, and CXP: collision cell exit potential | | | | | | | |
